# Three-Dimensional Reconstruction from Single Image Base on Combination of CNN and Multi-Spectral Photometric Stereo

**DOI:** 10.3390/s18030764

**Published:** 2018-03-02

**Authors:** Liang Lu, Lin Qi, Yisong Luo, Hengchao Jiao, Junyu Dong

**Affiliations:** College of Information Science and Engineering, Ocean University of China, Qingdao 266100, China; luliang@stu.ouc.edu.cn (L.L.); qilin@ouc.edu.cn (L.Q.); luoyisong@stu.ouc.edu.cn (Y.L.); jiaohengchao@stu.ouc.edu.cn (H.J.)

**Keywords:** depth estimation, convolutional neural network, multi-spectral photometric stereo

## Abstract

Multi-spectral photometric stereo can recover pixel-wise surface normal from a single RGB image. The difficulty lies in that the intensity in each channel is the tangle of illumination, albedo and camera response; thus, an initial estimate of the normal is required in optimization-based solutions. In this paper, we propose to make a rough depth estimation using the deep convolutional neural network (CNN) instead of using depth sensors or binocular stereo devices. Since high-resolution ground-truth data is expensive to obtain, we designed a network and trained it with rendered images of synthetic 3D objects. We use the model to predict initial normal of real-world objects and iteratively optimize the fine-scale geometry in the multi-spectral photometric stereo framework. The experimental results illustrate the improvement of the proposed method compared with existing methods.

## 1. Introduction

A major problem in computer vision is the sensing of structure and geometry of the three-dimensional world from the two-dimensional images. Compared with depth sensors, an image-based method has the advantages of lower equipment cost and easy acquisition of high-resolution data [1].

Image-based methods can be divided into two types: the active vision methods [2,3,4,5] and the passive vision methods [6,7,8,9]. Active vision-based method estimates the depth of field through the interaction of light and surface, such as shape from shading (SFS), photometric stereo (PS), and structured light (SL), etc. On the other hand, the method based on passive vision estimates the depth of field based on the principle of stereo geometry through matching clues among images, such as structure from motion (SFM). Many methods require a series of images (usually more than two), which limits the application in dynamic scenarios.

Photometric stereo is one of the most famous methods for 3D reconstruction, which requires that the position of the light source be changed while the relative position of the camera and the target is fixed. Drew et al. [5], Kontsevich et al. [10] and Woodham [11] first demonstrated the multispectral photometric stereo method, which can estimate the surface normal at each pixel, requiring the constant chromaticity of the surface and three spectrally and spatially separated light sources. Tsiotsios et al. [12] proved that three lights are enough to compute tridimensional information. Anderson et al. [13] used a more principled framework and proposed a color photometric stereo method without the need of a depth camera. Decker et al. [14] and Kim et al. [15] respectively analyzed the influence of varying chromaticity and proposed a time division multiplexing technology to relax the constraints of chromaticity consistency, which the traditional multi-spectral photometric stereo method requires. Meanwhile, Janko et al. [16] dealt with that problem by regularization of the normal field, avoiding the need for time multiplexing by tracking texture on the surface and optimizing both surface chromaticity and normal direction over a complete sequence. Hernandez et al. [17] presented an algorithm and the associated capture methodology to acquire and track the detailed 3D shape, bends, and wrinkles of deforming surface. Narasimhan et al. [18] presented a novel method to recover surface albedo, normal and depth map in scattering medium which requires a minimum of four images. Petrov [19] proposed a frequency division multiplexing method, through the target response to different frequencies of the light source for three-dimensional reconstruction, to overcome the traditional photometric stereo algorithm image acquisition process complex problem. Ma et al. [20] used a combination of structured light and time-multiplexed spherical illumination patterns to achieve high quality results.

Deep learning has made various breakthroughs in the field of computer vision. In particular, deep convolutional neural network (CNN) can handle many computer vision problems such as object detection, image segmentation, image classification, scene understanding and depth estimation. Recently, several methods for estimating depth using CNN have been proposed [21,22]. Most of them aim to estimate the depth of the scene, such as indoor living or outdoor streets. However, the depth estimates obtained by these depth-based methods are often coarse and cannot be used for high-precision requirements.

In this paper, we focus not only on the depth estimation of the entire scene, but also on the depth estimation of fine objects by combining deep CNN (DCNN) with multi-spectral photometric stereo. We use CNN to estimate the coarse depth from a single image, and then input it as an initialization input to photometric stereo for finer surface details. Due to the lack of data for multi-spectral photometric stereo, we synthesize color images by rendering models of the ShapeNet dataset and use the pre-trained network to estimate the depth of similar real world objects. The result of the depth estimation is used as input to the multi-spectral photometric stereo method and the surface normal map of the object can be calculated.

The following organization of this paper is as follows. In Section 2, we introduce the latest research progress on depth estimation from single image based on photometric stereo or deep learning. In Section 3, we elaborate our method, including the network structure, parameter setting and training data acquiring. Then we introduce the multi-spectral photometric stereo. Then, in Section 4, we present our experimental results, including the depth prediction of real world objects, and the reconstruction result of the proposed method.

## 2. Related Work

### 2.1. Photometric Stereo

Photometric stereo is one of the most effective methods in the field of image-based 3D reconstruction, which is highlighted by the high-resolution and fine reconstruction details [23]. A stationary camera captures a series of images (at least three) of a 3D object under multiple controlled illuminations. The intensity with the same image coordinate changes across these images with respect to the various directions of illuminations. Accordingly, the surface normal of this object can be computed based on corresponding intensities and lighting direction. The depth information is integrated by normal afterward, and then a fine detailed reconstruction of the object is obtained.

Photometric stereo is first introduced by Woodham [4]. He limited the method to the Lambertian surface reflectance model, which assumes that the albedo for each point on the object is constant. Coleman et al. [24], Nayer et al. [25], Lin et al. [26] and Jensen et al. [27] relaxed assumptions about non-Lambertian reflectance models such as the Bidirectional Reflectance Distribution Function (BRDF) [28] and the Bidirectional scattering-surface reflectance distribution function (BSSRDF) [27], etc. Several works dealt with the frequent presence of shadows and specular in an image (e.g., [29]). However, these methods suffer the same limitation that all images must be captured relative to the scene as the illumination changes. This means that three-dimensional light cannot reconstruct objects in motion.

To relax this restriction, Drew et al. [5] and Kontsevich et al. [10] initially proposed a multi-spectral photometric stereo technique, which can obtain a detailed geometry structure from a single image. In essence, multi-spectral photometric stereo is photometric stereo with colored light. Unlike photometric stereo which photographs objects under varying white lights and processes gray-scale images, the multi-spectral photometric stereo captures a RGB image, which stores pixels as one byte each for red, green, and blue values, under three colored light sources at one time. 

Commercial depth sensors such as Kinect and Real Scene can acquire three-dimensional information of objects in real time without the need to know objects or lighting in advance. The existing works use depth sensors to improve the depth estimation of luminosity. For example, Zhang et al. [30] and Yu et al. [31] introduced several sensor fusion schemes that combine active stereo with photometric stereoscopy. They block the Kinect’s quantification effect and enhance the surface detail. Moreover, their methods work well with changes in illumination with minimum intensity and ambient light conditions. However, these methods are highly dependent on the results of the depth sensor and require high computational costs. In addition, the resolution of current depth sensors is comparable to that of off-the-shelf digital cameras.

Although the traditional photometric three-dimensional method can achieve better results, the reconstruction process is still quite limited when this method is applied to estimate the surface depth of a single RGB image. Good results require ideal assumptions, additional system configuration, lots of calculation time and calibration of the lighting direction. In order to deal with these limitations, we propose a scheme to enhance the traditional photometric stereo through deep convolutional neural networks.

### 2.2. Machine Learning in Depth Estimation

Machine learning has made dramatic achievements in the field of computer vision. Many of the existing works are deeply estimated using machine learning methods. Eigen et al. [21] employed two (coarse and fine) deep network stacks to generate a coarse global estimation firstly and refined this estimation locally afterward. Liu et al. [22] formulated depth estimations into a continuous conditional random field learning problem, and presented a deep convolutional neural field model to solve the problem. Xiong et al. [32] apply dictionary learning to jointly optimize geometry and join constructs. It uses a triangular mesh to represent the surface of the object. However, the original dictionaries have to be given through dense point clouds, which means that their method is only used to refine the pre-reconstructed geometry. Recently, DCNN have attracted the attention of researchers in many fields compared with other machine learning methods. Deep CNN methods can estimate depth from a single image because of their ability to learn. This advantage allows DCNN to enhance traditional photometric methods with a single image rather than multiple images. Liu et al. [33] used a discriminatively-trained Markov Random Field (MRF) that incorporates multiscale local- and global-image features, and models both depths at individual points as well as the relation between depths at different points, to estimate depth from a single monocular image. Ladicky et al. [34] generalized the depth estimation and semantic segmentation as a multiple semantic classification problem. Yoon et al. [35] adopted a generative adversarial network (GAN) for fine-scale normal estimation using a single near-infrared (NIR) image. 

Tatarchenko et al. [36] predicted the depth map of RGB images using an encoder-decoder network. Mousavian et al. [37] proposed a new network, which uses the same loss function to fine tune through phase training, and realizes two functions of semantic segmentation and depth estimation. Other related studies include methods based on residual learning [38], regression to forests [39], multi-scale methods [40], conditional random fields [41], relative depth comments [42], two-streamed network [43], etc.

Although DCNN has a high learning ability, estimating the depth from a single image is still an unsuitable problem. The DCNN depth estimation is still not accurate enough in some applications. In addition, the huge demand for training data makes CNN more practical than the more traditional photometric methods. Unlike existing work, we combine depth CNN with multi-spectral PS for depth estimation. Therefore, our method can estimate depth information from a single image with higher precision.

## 3. Methods

In this section, the details of the proposed depth estimation scheme will be discussed. The scheme consists of two main parts: (a) a multi-spectral PS algorithm and (b) a deep convolutional neural network. The proposed method uses the multi-spectral photometric stereo to enhance the depth estimation from the deep convolutional neural network to reconstruct fine details.

### 3.1. Multi-Spectral Photometric Stereo

**Figure 1:**
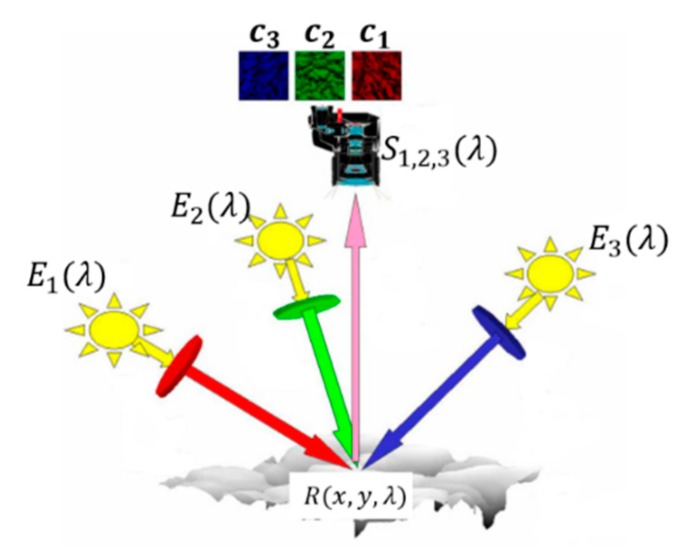
The illustration of multi-spectral photometric stereo.

The traditional multi-spectral photometric stereo technique can reconstruct the 3D geometry needing only a color image. The image should be obtained under the trichromatic light source with known angles, as shown in Figure 1.

The principle of multi-spectral photometric stereo is shown in Equation (1).

(1) $$c_{i}\left(x,y\right)=\underset{i}{\sum }l_{j}^{T}n\left(x,y\right)\int^{\text{​}}E_{j}\left(\lambda \right)R\left(x,y,\lambda \right)S_{i}\left(\lambda \right)d\lambda$$

where, *l_j_* is the *j*-th illumination direction vector, *n* (*x*, *y*) is the normal vector of a certain point of the target, *E_j_*(*λ*) is the illumination intensity, *R*(*x*, *y*, *λ*) is a parameter related with the albedo and chromaticity of a certain point of the target, and *S_i_*(*λ*) is the color response of the camera photosensitive element. 

Assume *R*(*x*, *y*, *λ*) as the product of *ρ*(*x*, *y*) and *α*(*λ*), which represent the albedo and the chromaticity respectively, then put all items which are related with *λ* as a whole, and we can get a parameter matrix *V*, as shown in Equation (2):

(2) $$V_{ij}=\int^{\text{​}}E_{j}\left(\lambda \right)\alpha \left(\lambda \right)S_{i}\left(\lambda \right)d\lambda$$

So we can rewrite Equation (1) as Equation (3), and obtain Equation (4):

(3) C=VLρn

(4) $$n=\frac{V^{-1}L^{-1}c}{\left\|V^{-1}L^{-1}c\right\|}$$

That is, the exact solution of the normal vector of the target surface can be obtained on the premise that the target’s chromaticity and the illumination direction are known.

The traditional multi-spectral photometric stereo algorithm has advantages such as it can reconstruct the 3D model only need one color image with a tricolor light source, so it can be used in video reconstruction problems, and it has high accuracy in horizontal and vertical directions. However, the quality of multi-spectral photometric stereo reconstruction algorithm’s result has great relationship with the initial depth value.

### 3.2. Deep Convolutional Neural Network

We build our network based on the simplest code–decode structure. By adding fully-connection layers and applying the dropout strategy before decoding, our network is established to estimate a global depth map from a single image.

#### 3.2.1. Architecture

**Figure 2:**
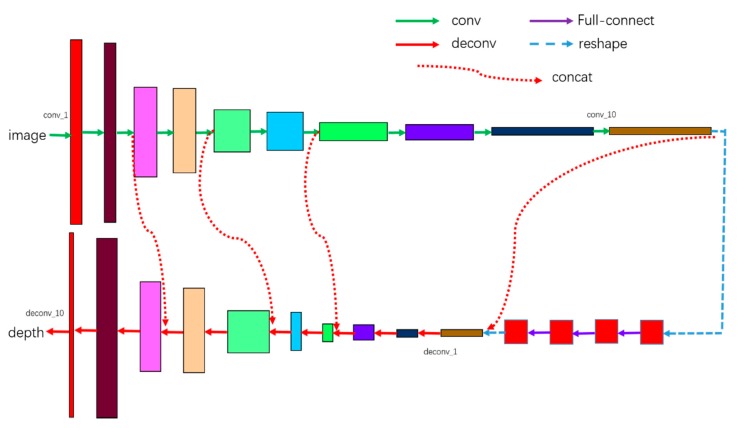
The architecture of our network. Conv is a convolution operation, and deconv is a deconvolution operation.

The architecture of the proposed network is shown in Figure 2. The network contains twenty-four layers, including ten convolution layers, four fully-connection layers, and ten deconvolution layers. We do not use any pooling strategy in our network. The details of our network are expounded in Table 1.

The values of column “weights” represent the size of convolution kernel and the strides, e.g., the value of conv_1’s weights is (5,5,2,2), which means the size of convolution kernel is 5 × 5, and the strides is (1,2,2,1). There are four fully-connection layers after ten convolution layers. Before the fully-connection operation, the output of conv_10 needs to be transformed into a vector. We use the dropout strategy in all four layers to improve the robustness, and the parameter keep_prob is set to 0.5. The output of the last full-connection layer should be transformed to a tensor and its shape should be the same as that of conv_10. The activate function for all convolution layers and de-convolution layers is Leaky ReLU non-linearity with the negative slope 0.2, except the last de-convolution layer, whose activate function is ReLU, since all the depth we would like to predict is positive. 

We use the L2 norm as the loss function to represent the difference between the network output and the ground truth.

#### 3.2.2. Training

The lack of data makes it difficult to train the network with a real object. We train our network with synthetic images rendered using the ShapeNet dataset [44]. The dataset contains 55 common object categories with about 51,300 unique 3D models. We render the 3D models based on a script on GitHub [45], which can render a 3D model to 2D images at different viewing angles with Blender. We’ve improved the script so that it can generate 2D images at different viewing angles for the same target illuminated by the red, green and blue light sources.

### 3.3. Combination of Deep Convolution Neural Network and Multi-Spectral PS

According to Equation (3), if we assume that there is a matrix M,

(5) M=VLρ

Then the surface normal of the object can be computed by

(6) $$n=M^{-1}C$$

Normally, the matrix M is calibrated by measuring the RGB response corresponding to each direction of the surface. However, this calibration process requires an additional specular sphere to estimate the light source direction with three image sequences. In this paper, we abandon this complex calibration process and use the output of the DCNN.

Local normal and intensities are known for 3 pixels with equal albedo. Therefore, if we can find these three pixels and its normal, the problem will be solved. The normal n may be calculated from the depth image generated by DCNN. Although the geometry obtained using DCNN is not accurate enough, there are still some valid depth pixels correctly estimated. We use the random sample consistency (RANSAC) algorithm to select those valid pixels and estimate the matrix M.

To achieve this assumption, the image is segmented into different super-pixels using a simple linear iterative clustering (SLIC) technique and it is assumed that each pixel in the same super-pixels has equal albedo and chromaticity. Using the estimated matrix M, a fine and detailed depth map can be obtained from a single RGB image with uncalibrated light sources.

## 4. Experiments

### 4.1. The Synthesis Dataset Rendered from ShapeNet

**Figure 3:**
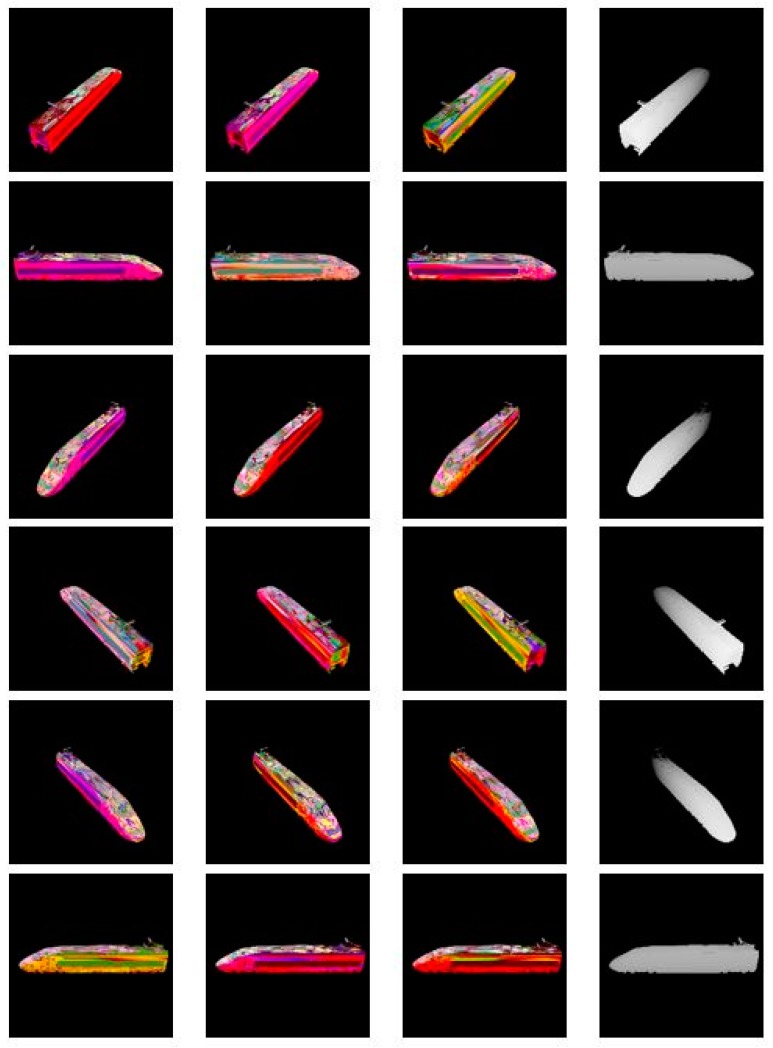
The images generated using the train model. The first three columns are RGB images and the last column is the depth image.

During the experiment, we fixed the camera at 12 different positions respectively and set the three light sources with 1320 different angle combinations. We obtained 15,840 rendered images of different colors with different angles. The size of each image is 600 × 600. At the same time, we also got the depth image corresponding to each image as the ground truth of the training network. We used 12,000 images as training data, and the remaining 3840 images as the test data. Some of the pseudo color images we generated used in the train model are shown in Figure 3. It should be noted that, because the depth data rendered is opposite to the actual meaning, the closer the position to the camera, the greater the brightness in the depth map.

### 4.2. Result of Our Network

#### 4.2.1. Experiment Results

We use Tensorflow (https://storage.googleapis.com/tensorflow/linux/gpu/tensorflow-0.8.0-cp27-none-linux_x86_64.whl) with the Nvidia GT730 graphics card (Beijing, China) to implement and train the proposed network. The training process uses a size of 16 batches. The loss function is optimized using the Adagrad Optimizer and the learning rate is 0.001. We initialize the weights with a zero-mean Gaussian distribution and a standard deviation of 0.02. 

**Figure 4:**
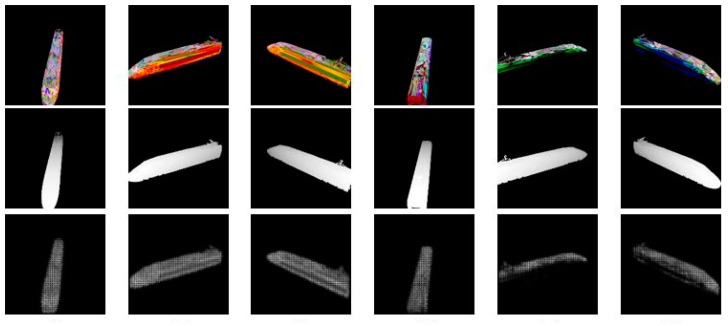
The results using the same train model. The top line shows the test images, the middle line shows the ground truth of each image, and the bottom line shows the depth our network predicts.

**Figure 5:**
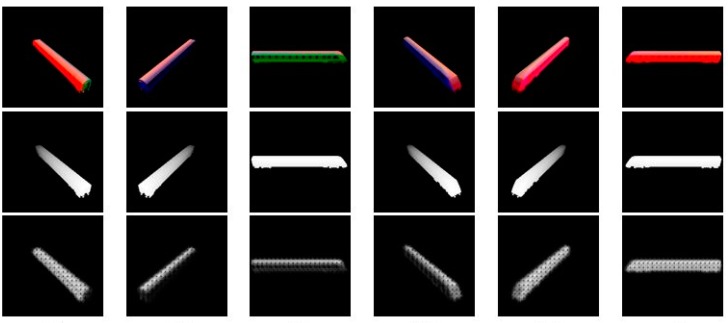
The results using a new train model. The top line shows the test images, the middle line shows the ground truth of each image, and the bottom line shows the depth our network predicts.

For testing the robustness of our network, we have generated two kinds of test set, the first one is images generated with the same train model which we used to generate the train dataset, at different viewing angles, and the second one is images generated with a new train model of ShapeNet. The results we got after 40,000 iterations are shown in Figure 4 and Figure 5 respectively.

#### 4.2.2. Quantitative Analysis

The quantitative analysis of the results above is shown in Table 2. Suppose the image has N valid points, $d_{i}^{*}$ is the ground truth depth of the *i*-th point, and *d_i_* is the prediction depth of the *i*-th point using our network. The meaning of each parameter in the table is:

- Mean relative error (rel), which can be calculated according to Equation (7):
  (7) $$\frac{1}{N}\underset{i}{\sum }\frac{\left|d_{i}-d_{i}^{*}\right|}{d_{i}^{*}}$$
- Root mean squared error (rms), which can be calculated according to Equation(8):
  (8) $$\sqrt{\frac{1}{N}\underset{i}{\sum }{\left(d_{i}-d_{i}^{*}\right)}^{2}}$$
- Accuracy with threshold t (δ), this is a statistical parameter that is used to count the percentage of pixels matching a certain condition in the image with respect to the total number of pixels in the image. According to the different values of t, the result is divided into three grades, that is, when t is 1.25, the result is δ1, when t is 1.252, the result is δ2, and when t is 1.253, the result is δ3. It can be calculated according to Equation (9):
  (9) $$\delta =\frac{1}{N}\underset{i}{\sum }\eta_{i}\;\eta_{i}=\{\begin{array}{c} 1\;\;\;if\;T<t\\ 0\;\;\;if\;T\geq t \end{array}\;T=\max\left(\frac{d_{i}}{d_{i}^{*}},\frac{d_{i}^{*}}{d_{i}}\right),\;t\in \left[1.25,\;1.25^{2},1.25^{3}\right]$$

### 4.3. Result of Combination of Deep Convolution Neural Network and Multi-Spectral PS

#### 4.3.1. Experiment Results

We use the result of our network as the initial depth estimate and optimize it with multi-spectral photometric stereo. Our approach is to test with real objects, including toy aircrafts, gypsum boats and plastic trains. Each object is captured as a single image under the trichromatic light source.

**Figure 6:**
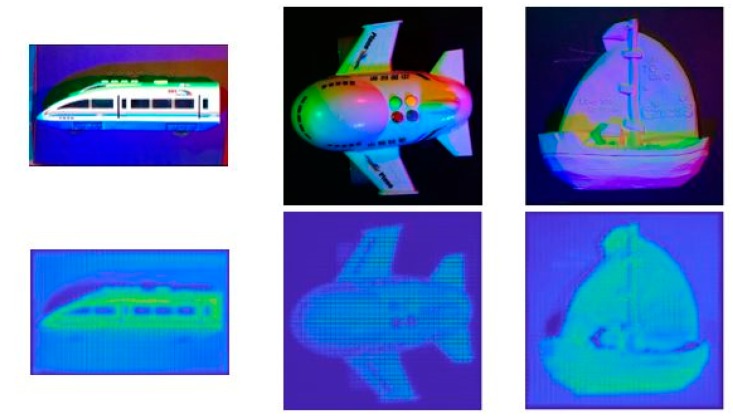
Results of our network with real world objects. The top line shows the input images, the bottom line shows the estimated depth result from our network respectively, which is produced by Matlab’s imagesc function.

We tested down sampled images of our network with plasterboard to 600 × 600 size. We first estimate the depth map, and then combine the final result with multispectral luminosity. The depth estimated by our network is shown in Figure 6.

Figure 6 shows the depth estimation generated by our network. It can be found from the figure that our depth prediction results include a bar divider, and it looks a bit vague because our padding parameter for DCNN chose ‘valid’, and we performed multiple convolutions. Although the result did not contain enough detail as in the real object, it still produced a good shape and profile.

**Figure 7:**
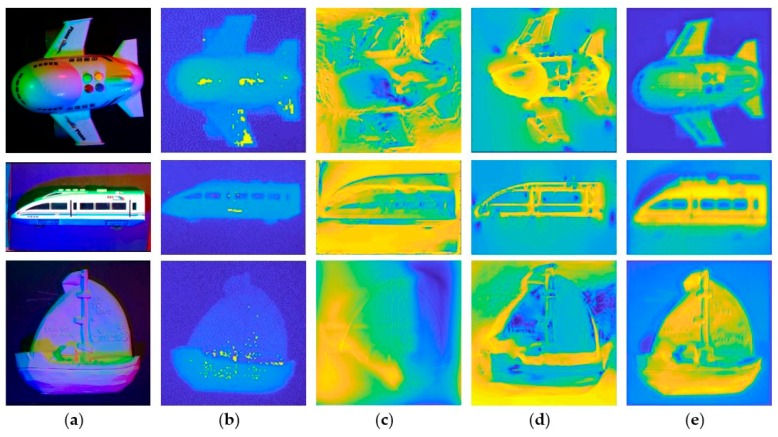
The final results produced by Matlab’s imagesc function. (**a**) The input images. (**b**) The outputs of Kinect. (**c**) The result of the depth estimation of traditional multi-spectral PS. (**d**) The result of [10]. (**e**) The result of the depth estimation of our method.

Figure 7 shows the results of our method compared to the results of Kinect, the results of traditional multispectral PS, and the results of [10] with continuous CRF. In order to facilitate the error analysis, we adjust the depth data of all images to [0, 1]. Compared with Kinect, our method has a higher resolution and fewer holes. Compared with the results of [10], our results allow for finer detail and more accurate depth estimation.

#### 4.3.2. Quantitative Analysis

There is a large amount of noise (i.e., the black spots in the image) in the depth image obtained by KINECT (Microsoft, Redmond, Washington D.C., USA), using it as the ground truth depth without any procession will lead to great errors. Therefore, we firstly perform median filtering and hole filling on the depth image obtained by KINECT, and obtain the approximate ground truth depth images.

**Figure 8:**
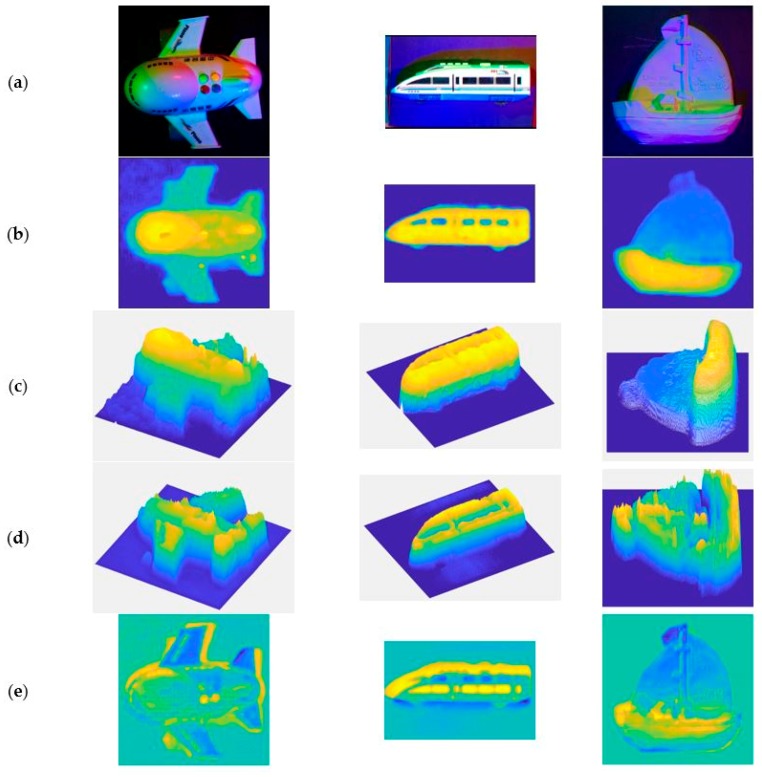
(**a**) The input image. (**b**) The approximate ground truth depth images after processing. (**c**) The 3D representation of (**b**). (**d**) The 3D representation of the result of our deep convolutional neural network (DCNN) and multi-spectral photometric stereo (DCNN+MS-PS) method. (**e**) The error map between (**c**,**d**).

Figure 8 shows the results of the pretreatment, as well as the 3D representation of them and the results obtained by our DCNN and multi-spectral photometric stereo (DCNN+MS-PS) method.

Table 3 shows the quantitative analysis of the depth estimate results of our network (DCNN), traditional MS-PS, and combination of DCNN and MS-PS.

As can be seen from Table 3, the proposed method yields better results than using DCNN or MS-PS alone for the parameter δ. Because δ is a parameter that measures the accuracy of the reconstructed result from a statistical point of view, that is, our method can increase the number of points which are closer to the true value in the predicted result.

However, our method is not very good at improving the result for both ‘rel’ and ‘rms’ parameters. This may be caused by a variety of factors, such as the target’s color, material, and light conditions.

For example, in the aircraft image, there are four differently colored buttons on the rear of the aircraft, whose height should be slightly above the aircraft's fuselage. However, in the reconstruction result of our method, this part shows three deep pits and a shallow one, that is, the button part has not been reconstructed correctly. Another example is, in the train image, the depth of a train’s window should have been about the same depth as the train’s shell, but the depth predictions at the corresponding position of the train window are significantly incorrect due to the different materials and colors. 

For the ship image, the main reason for the huge deviation is the uneven illumination. From the RGB images, we can see that there is a yellowish and green area in the lower part of the image. The difference between the prediction results of this part and the ground truth value leads to the error for ‘rel’ and ‘rms’ parameters.

## 5. Conclusions

Three-dimensional reconstruction from single color image with unknown illumination is a challenging problem, because it is affected by many factors such as the structure of the object, surface albedo, the frequency and direction of incident light, and the viewing angle, etc. Deep learning can be viewed as an end-to-end optimization process with massive parameters, and theoretically, we can use these parameters to simulate the effect of these factors in the imaging process to solve this ill-posed problem.

We proposed a new method for 3D reconstruction from a single image, and it mainly focuses on three aspects. Firstly, we built a depth-estimate network based on code–decode structure and obtained a rough depth map. Second, we investigated the use of synthetic pseudo-artifact color images to train the network. In this way, a large number of labeled data can be obtained. Thirdly, we combined the depth prediction result produced by our network with the traditional multi-spectral photometric stereo algorithm, and we obtained accurate 3D information of the object with a resolution as high as the digital camera used for photometric stereo.

## Figures and Tables

**Table 1:** Details of our deep convolution neural network (DCNN). Conv is a convolution operation, and deconv is a deconvolution operation.

Name	Input	Weights	Output Layers	Remarks
conv_1	image	(5,5,2,2)	32	padding=’VALID
conv_2	conv_1	(5,5,1,1)	32	padding=’VALID
conv_3	conv_2	(5,5,2,2)	64	padding=’VALID
conv_4	conv_3	(5,5,1,1)	64	padding=’VALID
conv_5	conv_4	(5,5,2,2)	128	padding=’VALID
conv_6	conv_5	(5,5,1,1)	128	padding=’VALID
conv_7	conv_6	(5,5,2,2)	256	padding=’VALID
conv_8	conv_7	(5,5,1,1)	256	padding=’VALID
conv_9	conv_8	(5,5,2,2)	256	padding=’VALID
conv_10	conv_9	(5,5,1,1)	256	padding=’VALID
reshape	reshape conv_10 to 1×N			
fc_1	conv_10	N×4096	/	keep_prob=0.5
fc_2	fc1	4096×4096	/	keep_prob=0.5
fc_3	fc2	4096×4096	/	keep_prob=0.5
fc_4	fc3	4096×N	/	keep_prob=0.5
reshape	reshape fc_4 to the shape of conv_10			
deconv_1	fc4+conv10	(5,5,1,1)	128	padding=’VALID
deconv_2	deconv_1	(5,5,2,2)	64	padding=’VALID
deconv_3	deconv_2	(5,5,1,1)	64	padding=’VALID
deconv_4	deconv_3+conv_7	(5,5,2,2)	32	padding=’VALID
deconv_5	deconv_4	(5,5,1,1)	32	padding=’VALID
deconv_6	deconv_5+conv_5	(5,5,2,2)	16	padding=’VALID
deconv_7	deconv_6	(5,5,1,1)	16	padding=’VALID
deconv_8	deconv_7+conv_3	(5,5,2,2)	8	padding=’VALID
deconv_9	deconv_8	(5,5,1,1)	8	padding=’VALID
deconv_10	deconv_9	(5,5,2,2)	1	padding=’VALID

**Table 2:** The quantitative analysis of the results.

Image	rel ^1^	rms ^1^	δ_1_^2^	δ_2_^2^	δ_3_^2^
Figure 4a	0.5935	0.5083	0.0672	0.2120	0.4736
Figure 4b	0.5738	0.5083	0.0310	0.2215	0.5116
Figure 4c	0.5836	0.5070	0.0693	0.2339	0.4447
Figure 4d	0.6497	0.6010	0.0410	0.1205	0.2748
Figure 4e	0.8300	0.7292	0.0167	0.0591	0.1224
Figure 4f	0.7302	0.6282	0.0133	0.0771	0.2094
Figure 5a	0.4225	0.2899	0.3824	0.6693	0.7788
Figure 5b	0.6329	0.5032	0.1700	0.3004	0.3848
Figure 5c	0.6829	0.6607	0.0381	0.1571	0.2502
Figure 5d	0.6358	0.4792	0.1272	0.2744	0.4166
Figure 5e	0.4741	0.2979	0.3527	0.6156	0.7533
Figure 5f	0.3473	0.3353	0.1949	0.6677	0.8589

^1^ lower is better, ^2^ higher is better.

**Table 3:** The quantitative analysis of the results of Figure 7. MS-PS is an acronym for multi-spectral photometric stereo, and the parameter ‘rel’ and ‘rms’ are defined in Equations (7) and (8).

Image		rel ^1^	rms ^1^	δ_1_^2^	δ_2_^2^	δ_3_^2^
aircraft	DCNN	0.5716	0.2928	0.1262	0.2667	0.4165
	MS-PS	2.6899	0.6273	0.5221	0.5746	0.6315
	DCNN+MS-PS	2.8001	0.4118	0.5832	0.7078	0.8007
train	DCNN	0.8165	0.5204	0.2237	0.3637	0.4502
	MS-PS	1.8237	0.7501	0.0607	0.0915	0.1266
	DCNN+MS-PS	0.7368	0.5831	0.2300	0.4407	0.4979
ship	DCNN	2.1245	0.3684	0.2371	0.3756	0.4887
	MS-PS	1.1393	0.3393	0.1560	0.2689	0.3621
	DCNN+MS-PS	1.2453	0.3089	0.2184	0.4048	0.5548

^1^ lower is better, ^2^ higher is better.
